# Assessing Interventions against Coronavirus Disease 2019 (COVID-19) in Osaka, Japan: A Modeling Study

**DOI:** 10.3390/jcm10061256

**Published:** 2021-03-18

**Authors:** Ko Nakajo, Hiroshi Nishiura

**Affiliations:** 1 Kyoto University School of Public Health, Yoshida-Konoe-cho, Sakyo-ku, Kyoto 606-8501, Japan; koudylan@gmail.com; 2 Graduate School of Medicine, Hokkaido University, Kita 15 Jo Nishi 7 Chome, Kita-ku, Sapporo-shi, Hokkaido 060-8638, Japan; 3 Sanofi K.K. Tokyo Opera City Tower, 3-20-2, Nishi Shinjuku, Shinjuku-ku, Tokyo 163-1488, Japan

**Keywords:** epidemiology, severe acute respiratory syndrome coronavirus 2 (SARS-CoV-2), coronavirus disease 2019 (COVID-19), public health, control, mathematical model

## Abstract

Estimation of the effective reproduction number, *R*(*t*), of coronavirus disease (COVID-19) in real-time is a continuing challenge. *R*(*t*) reflects the epidemic dynamics based on readily available illness onset data, and is useful for the planning and implementation of public health and social measures. In the present study, we proposed a method for computing the *R*(*t*) of COVID-19, and applied this method to the epidemic in Osaka prefecture from February to September 2020. We estimated *R*(*t*) as a function of the time of infection using the date of illness onset. The epidemic in Osaka came under control around 2 April during the first wave, and 26 July during the second wave. *R*(*t*) did not decline drastically following any single intervention. However, when multiple interventions were combined, the relative reductions in *R*(*t*) during the first and second waves were 70% and 51%, respectively. Although the second wave was brought under control without declaring a state of emergency, our model comparison indicated that relying on a single intervention would not be sufficient to reduce *R*(*t*) < 1. The outcome of the COVID-19 pandemic continues to rely on political leadership to swiftly design and implement combined interventions capable of broadly and appropriately reducing contacts.

## 1. Introduction

The coronavirus disease (COVID-19) has continued to cause significant morbidity and mortality worldwide. Although specific treatments have been developed and approved in some countries, the epidemic has produced an enormous surge in case loads and the demand for medical services [1,2]. There remains an unmet public health need in terms of the effective and prompt control of this pandemic. The development of specific interventions (e.g., efficacious vaccines that confer long-lasting protective immunity) might take months or even years. Thus, public health and social measures (PHSM, i.e., countermeasures that are conducted by individuals, institutions, communities, local and national governments and international bodies to suppress or contain the spread of an infectious disease, which was previously referred to as non-pharmaceutical interventions), including early case identification and quarantine of close contacts, social distancing, and potentially, population-wide lockdowns of cities, remain the primary control measures available.

As part of the evaluation of PHSM, the effective reproduction number (*R*(*t*), the actual average number of secondary cases per primary case at calendar time *t*), has been frequently employed [3,4,5,6,7,8,9,10]. Because *R*(*t*) reflects the frequency of transmission over time, it has been recognized as epidemiologically useful in assessing transmission dynamics in populations with varying susceptibility levels and experiencing various public health interventions. Published studies have estimated the *R*(*t*) of COVID-19 and assessed the impact of PHSM on *R*(*t*) at the country or city/district levels [3,4,11,12,13].

Despite the usefulness of *R*(*t*), it does not offer a threshold for vaccination, and moreover, it remains challenging to accurately estimate *R*(*t*) in real-time. Doing so would allow us to describe epidemic dynamics based on readily available data, and to assess the relevance of *R*(*t*) in evaluating PHSM. Theoretically, the estimation of *R*(*t*) should be based on the incidence of infection and the generation time (i.e., the time from infection in the primary case to infection in the secondary case) [14,15,16]. However, COVID-19 infection events are generally not directly observable, and full datasets are rarely available. As a result, most studies have leveraged information on illness onset or the serial interval (i.e., the time from illness onset in the primary case to illness onset in the secondary case), or both, to estimate *R*(*t*). While the estimation of *R*(*t*) based on illness onset is conventional, accumulating evidence suggests that pre-symptomatic transmission contributes to the secondary transmission of COVID-19 [17,18,19,20]. Estimates of *R*(*t*) based on illness onset data seldom take into account pre-symptomatic transmission.

To address the abovementioned challenges, we propose here a novel method to estimate *R*(*t*) as a function of the date of infection using the observable data of illness onset. We applied this method to epidemiological data from the Osaka prefecture, Japan, the prefecture with the third largest population after Tokyo and Kanagawa. The incidence of COVID-19 in Osaka was second only to that in Tokyo, and for this reason, the governor has frequently made announcements via mass media. We assessed the relationship between trends in *R*(*t*) and the implementation of PHSM.

## 2. Methods

### 2.1. Epidemiological Data

In Japan, COVID-19 cases are confirmed by reverse transcriptase polymerase chain reaction. All confirmed cases are notified to the government. We analyzed the date of illness onset of COVID-19 cases in the Osaka prefecture. The capital city, Osaka city, is the most populous ordinance-designated city in the prefecture (approximately 2.7 million inhabitants). In the Osaka prefecture, the index COVID-19 case was reported on 29 January 2020 and a total of 11,249 cases had been reported as of 13 October 2020. We analyzed 8818 cases for which information of illness onset by 28 September 2020 was available. Because it was likely that no secondary cases were produced by the index case given the time window of illness onset from 29 January to 17 February (i.e., the day of illness onset for the second case identified), we used 17 February as day 0 in subsequent analyses.

### 2.2. Public Health Intervention Implementation

Several PHSMs were implemented in the Osaka prefecture throughout the period of our investigation (Table 1). Before the declaration of a state of emergency by the Japanese government on 7 April, the governor had already requested Osaka residents in late March to voluntarily abstain from weekend social activities and to refrain from going to bars and nightclubs. When the first wave neared its end, the prefecture announced the criteria for its alert system, referred to as the “Osaka model,” to confront future epidemics. The first alert was issued on 12 July and was followed by sequential requests for the voluntary restriction of social behaviors and the closing of shops/bars in specified areas of Osaka city.

### 2.3. Model Descriptions

To estimate *R*(*t*), we employed a renewal process model. If *j*(*t*) represents the incidence of infection at calendar time t, the commonly used renewal equation is:

(1) $$j\left(t\right)=R\left(t\right)\int_{0}^{\infty }j\left(t-s\right)g\left(s\right)ds$$

where *g*(*s*) is the probability density function of the generation time *s*. A practical issue for estimating *R*(*t*) from Equation (1) is that *j*(*t*) must be known. However, in infectious disease epidemiology, *j*(*t*) is not directly observable. For this reason, our analyses of COVID-19 epidemiology prior to this study back-calculated *j*(*t*) based on the incidence of illness onset, *c*(*t*), which is readily observable. Assuming that the density function of the incubation period is known and that the incubation period is independently and identically distributed, the daily incidence of infection could be non-parametrically back-projected. While that procedure was theoretically elegant, the estimation process for *R*(*t*) required two steps of inference (i.e., back-calculation and solving the renewal equation) and was not statistically rigorous.

In the present study, we avoided this issue by adhering to the date of illness onset while estimating *R*(*t*) based on the date of infection (Nakajo and Nishiura, submitted). Here, we describe the improved renewal equation briefly. Assuming that the relative frequency of secondary transmission with respect to disease-age (i.e., the time since illness onset) *u*, *λ*(*u*), is known, *j*(*t*), the daily incidence of infection at calendar time *t* can be written as follows using the daily number of new illness onsets, *c*(*t*):

(2) $$j\left(t\right)=R\left(t\right)\int_{-x}^{\infty }c\left(t-u\right)\lambda \left(u\right)du$$

where *R*(*t*) is the instantaneous measure as a function of the date of infection, and pre-symptomatic secondary transmission is assumed to take place from *x* days prior to illness onset. Let the probability density function of the incubation period be *f*(*τ*). The relationship between the daily number of new illness onsets and the daily incidence of infection can then be described as:

(3) $$c\left(t\right)=\;\int_{0}^{\infty }j(t-\tau )f\left(\tau \right)d\tau$$

This equation is commonly used for back-calculating *j*(*t*) from *c*(*t*). Rather than doing so, here we substituted *j(t*) in the right-hand side of Equation (3), as calculated in Equation (2). Then, the relationship between the daily number of new illness onsets at time *t* and the effective reproduction number *R*(*t*) is described by:

(4) $$c\left(t\right)=\;\int_{0}^{\infty }R\left(t-\tau \right)\int_{-x}^{\infty }c\left(t-\tau -u\right)\lambda \left(u\right)f\left(\tau \right)dud\tau$$

Equation (4) can describe the epidemic dynamics using observable data by producing *c*(*t*) (i.e., the epidemic curve drawn by date of illness onset). This approach enabled us to estimate *R*(*t*) as a function of the date of infection, *t*, using information on observable illness onset. For the relative frequency of secondary transmission at disease-age *u*, *λ*(*u*), we used a gamma distribution with the peak at symptom onset and 12.3 days as the starting point for infectiousness; these values were estimated from 77 transmission pairs [18]. For the probability density of the incubation period, *f*(*τ*), we used a lognormal distribution with a mean of 5.2 days, estimated from 425 patients in Wuhan [21], consistent with the results of Linton et al. [22]. We assumed that (i) the probability density function of the incubation period and the frequency of secondary transmission relative to disease-age were independently and identically distributed, and (ii) the heterogeneity of transmission, including age dependence and spatial dependence, can be ignored. Moreover, we assumed that the extent of underreporting (i.e., ascertainment bias) remained unchanged over time.

The expected value of the daily incidence (i.e., the number of new illness onsets) was modeled by discretizing Equation (4) and changing the lower limit of the integration range in the second convulsion to zero, as follows:

(5) $$E(c_{t})=\overset{t}{\underset{\tau =0}{\sum }}R_{t-\tau }f_{\tau }\overset{t-\tau +x}{\underset{v=0}{\sum }}c_{t-\tau +x-v}\lambda_{v-x}.$$

where *f_τ_* is now discrete and referred to as the probability mass function of the incubation period. Assuming that the daily number of reported cases follows a Poisson distribution, the likelihood function to estimate *R_t_* is:

(6) $$\underset{t}{\prod }\frac{{\left(\sum_{\tau =0}^{t}R_{t-\tau }f_{\tau }\sum_{v=0}^{t-\tau +x}c_{t-\tau +x-v}\lambda_{v-x}\right)}^{c_{t}}\exp[-\sum_{\tau =0}^{t}R_{t-\tau }f_{\tau }\sum_{v=0}^{t-\tau +x}c_{t-\tau +x-v}\lambda_{v-x}]}{c_{t}!}$$

We used a piecewise constant model for *R*(*t*) that changes its value every 5 days [23]. This 5-day period was specifically chosen because it is in the range of published estimates of the serial interval for COVID-19 [17,18,20,24]. Maximum likelihood estimates of *R*(*t*) were obtained by minimizing the negative logarithm of Equation (5). To quantify the confidence intervals (CIs) of *R*(*t*), we implemented parametric bootstrapping using the Hessian matrix H. We obtained 1000 resamples of parameters from the normal distribution with mean ***θ***_0_ and standard deviation ***σ*,** equal to the square root of diagonal elements of the inverse Hessian matrix (***σ***^2^ = diag(H^−1^(***θ***_0_))). For each identical set of parameters, we assessed the potential variation in estimated parameter values. By taking the 2.5th and 97.5th percentiles of the simulated distributions, we obtained 95% CIs for *R*(*t*). The 95% CI for incidence was also computed using parametric bootstrapping.

As part of the validation process, we compared our estimates of *R*(*t*) with ones from a renewal equation model, in which the distributions of generation intervals and daily incidence obtained by back-calculation are convoluted. Specifically, the original method back-calculated the incidence of infection using the non-parametric back-projection method [25]. Subsequently, the following renewal process model was used:

(7) $$E\left(j\left(t\right)\right)=R\left(t\right)\int_{0}^{\infty }j\left(t-\tau \right)g\left(\tau \right)\frac{F\left(T-t\right)}{F\left(T-t+\tau \right)}dt$$

where *j*(*t*) is the back-calculated incidence of infection, *g*(*τ*) is the probability density function of the generation interval, *F*(.) is the cumulative distribution function of the time delay from infection to reporting, and *T* is the latest calendar time of observation. As real-time evaluation has been underway using back-calculated incidence of infection, the renewal Equation (7) involved adjustment for reporting delay. However, the structure is in principle comparable to the commonly used renewal equation, as well as Equation (2).

Using Equations (5) and (7), two different sets of *R*(*t*) estimates were obtained and overlaid with epidemic curves to assess their responsiveness to the implemented PHSM. We even included broad announcements by the governor of Osaka as PHSM, because they could have substantially reduced numbers of high-risk contacts. We calculated the coverage of the proposed Equation (5) in terms of maximum likelihood estimates from Equation (7) to assess the similarity between these equations throughout the epidemic.

### 2.4. Model Comparison and Sensitivity Analysis

To understand if *R*(*t*) estimates were associated with the important PHSM events described in Table 1, we compared the negative log likelihood values of all possible combinations of “event-based” models using step functions to approximate *R*(*t*). First, we computed a full model that accounted for all event dates affecting *R*(*t*) on days 31, 39, 43, 46, 94, 162, 165, 186 and 196. Among these dates, PHSMs were implemented on days 31, 39, 43 and 46 (first wave) and days 162 and 165 (second wave). To understand the importance of interventions, we carried out three different types of analyses. First, comparisons between the full and alternative models were made to account for the loss of known event dates. In the first wave, there were four event dates (day 31, 39, 43 and 46) and 15 possible combinations of those dates to be considered. In the second wave, there were two event dates (day 162 and 165) and three combinations (i.e., on–off, off–on, and off–off models). Likelihood ratio tests were performed. Because of the need to conduct multiple comparisons, Bonferroni correction of the global alpha level was performed. The desired alpha level (0.05) was divided by the number of comparisons in each wave (15 and 3, respectively), and *p*-values smaller than this reduced value were considered significant. Second, a joinpoint segmented regression model was used to assess whether a significant change in *R*(*t*) during each wave was associated with any of the start dates of key interventions [26]. This method enables the identification of inflection points (“joinpoints”) within trends over a specific period by employing permutation tests for model selection. Third, we estimated the relative reduction in the effective reproduction number associated with these dates. The relative reproduction number was estimated following each event date.

For baseline estimation, the 5-day period for the piecewise constant model of *R*(*t*) was chosen to keep the inference procedure simple and tractable, without requiring a statistical smoothing procedure. As a part of the sensitivity analyses, we modeled *R*(*t*) using 3-day and 4-day periods instead of the 5-day period. Using these different models, we compared the dates on which the *R*(*t*) took values below 1 for the first time during each wave.

### 2.5. Data Sharing Statement

The epidemiological data analyzed in this study are publicly available (https://covid19-osaka.info/; Accessed on 17 March 2021) and also downloadable from the online supplementary data. The R code used for estimating *R*(*t*) is available in the online supplementary text.

## 3. Results

**Figure 1:**
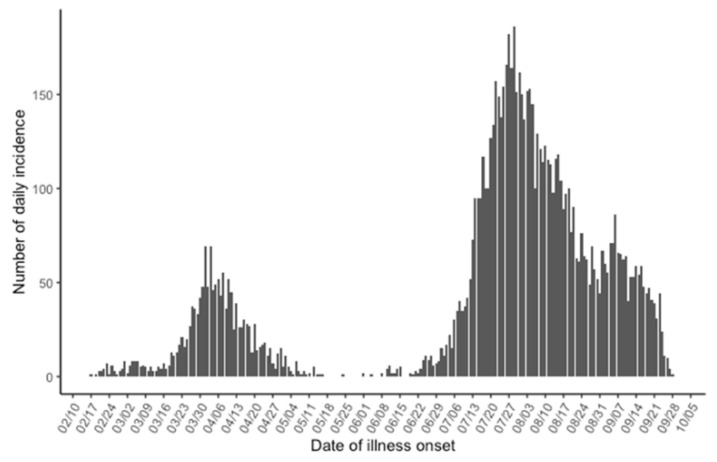
Daily numbers of new coronavirus disease 2019 (COVID-19) cases in Osaka prefecture from 17 February to 28 September 2020. Cases were counted as a function of the date of illness onset.

Figure 1 shows the epidemic curve as a function of the date of illness onset. There were two distinct epidemic waves, the first occurring from February to May and the other from June to October and onwards. The highest daily incidence during the first wave was 69 cases on 1 and 3 April, and the highest daily incidence during the second wave was 186 cases on 29 July. Because of delays in diagnosis and reporting, the date of reporting was delayed by 5–7 days on average from the actual real-time epidemic data. It should also be noted that the second wave has not smoothly declined over time: the rate of decline stagnated from late August, and a second hump with peak daily incidence of 86 cases was observed on 6 September 2020.

**Figure 2:**
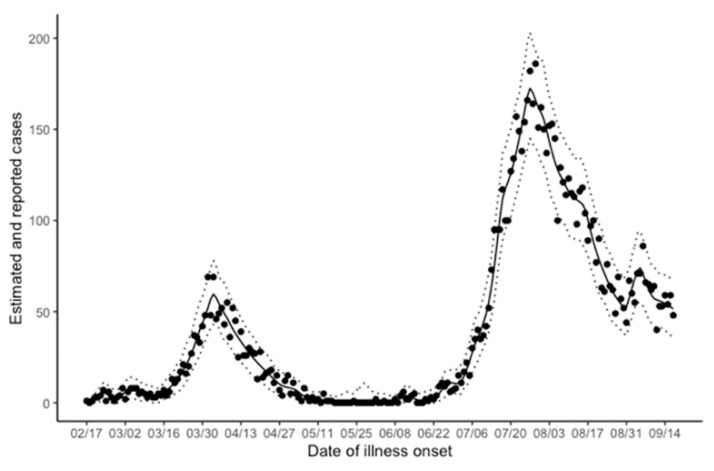
Comparison between the observed and model-predicted incidence of coronavirus disease 2019 (COVID-19) in Osaka prefecture. Comparisons were made as a function of the date of illness onset. Solid circles represent observed cases, while the continuous black line shows the maximum likelihood estimate of predicted incidence. Dotted lines represent the lower and upper boundaries of the 95% confidence intervals based on the bootstrap method.

Figure 2 compares our model predictions and associated 95% CIs using the bootstrap method against the observed epidemic curve. Our model qualitatively effectively reproduced the overall observed pattern of the epidemic. The model estimated the cumulative number of cases as 8520 by the end of the investigation period, whereas there were actually 8818 cases observed. The 95% CI for the total epidemic size ranged from 6367 to 11,066 cases.

**Figure 3:**
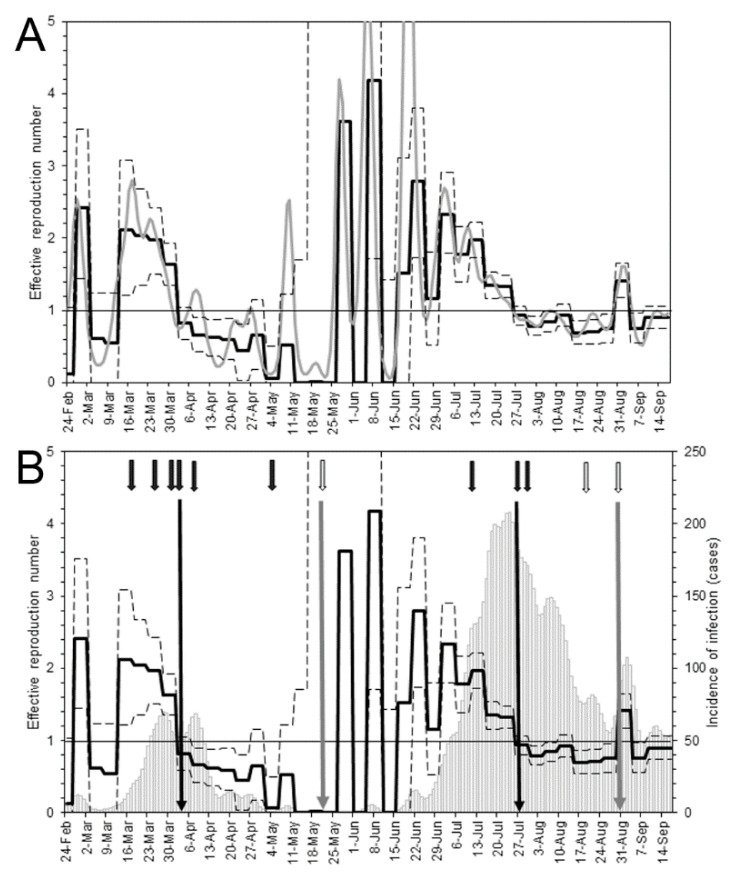
Estimation of the effective reproduction number, *R*(*t*), using two methods. (**A**) Comparison of *R*(*t*) estimates calculated using a novel method based on the observed date of illness onset (continuous black line) and using the existing method based on non-parametrically back-projected incidence of infection. Dashed lines show the 95% confidence intervals of *R*(*t*) based on the bootstrap method. The horizontal gray line indicates *R*(*t*) = 1; below this value incidence declines. (**B**) Chronological relationship between announcements in Osaka and *R*(*t*). Black arrows represent announcements of requests to reduce contacts or any other announcements associated with infection control. White arrows represent announcements of the cessation of specific countermeasures. Long black line shows when *R*(*t*) decline below 1, and long grey arrows indicate when reopening was declared. On 3 April, the governor requested voluntary restrictions on weekend outings. On 28 July, the governor requested residents to voluntarily abstain from social events involving the consumption of alcohol by five or more persons. On 21 May, Osaka was released from the state of emergency, and on 31 August, the governor permitted social events involving the consumption of alcohol by five or more persons. The overlaid bar chart shows incidence by the estimated date of infection.

The maximum likelihood estimates of *R*(*t*) are shown in Figure 3A. During the first wave, the effective reproduction number reached <1 (0.8; 95% CI: 0.6–1.0) for the first time on 2 April. The *R*(*t*) then remained <1 until 26 May, indicating that the epidemic was under control during this period. Subsequently, the *R*(*t*) showed an explosive oscillating pattern and increased until 26 July, when it again dropped to <1 (0.9; 95% CI: 0.8–1.0). Compared with the *R*(*t*) estimates based on the back-projected incidence of infection, our estimates revealed very consistent patterns. Of the 207 days of the observation period, the daily *R*(*t*) based on the estimated incidence of infection was contained within the 95% CI of our estimate for 141 days (68.1% of days examined).

Figure 3B shows the relationship between *R*(*t*) dynamics and the start and end dates of key PHSMs implemented throughout the epidemic. Our estimates of *R*(*t*) responded well to the implementation of each PHSM. During the first wave, successive voluntary restrictions were requested by the governor starting on 19 March. Subsequently, the *R*(*t*) decreased step-by-step in response to these interventions. On 3 April, voluntary restrictions on weekend outings were requested for the second time, and subsequently the *R*(*t*) declined to <1. Osaka was released from the state of emergency on 21 May, and 6 days later the *R*(*t*) showed multiple humps, indicating the beginning of the second wave. The *R*(*t*) declined to <1 again from 26 July, during which time voluntary restrictions on social events involving the consumption of alcohol by five or more persons were requested by the governor of Osaka. This request was ended on 31 August, and following this date the *R*(*t*) again sporadically reached >1.

**Figure 4:**
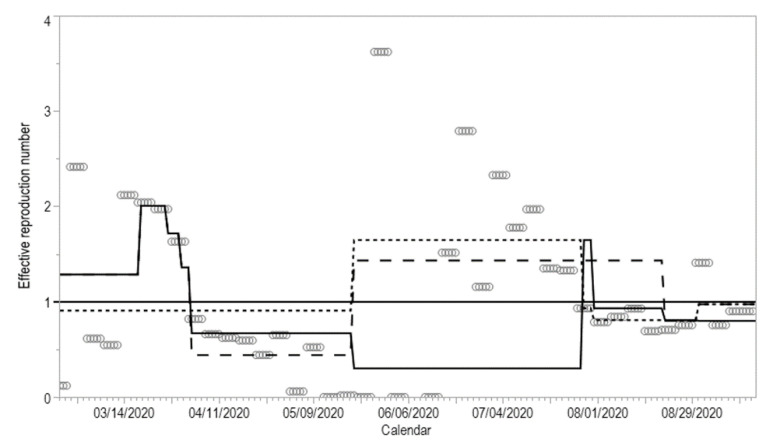
Comparison of event-based models of the effective reproduction number. Unfilled circles represent estimates based on the 5-day piecewise constant model. The solid line shows the full event-based model accounting for all changes in *R*(*t*) on days 31, 39, 43, 46, 94, 162, 165, 186 and 196. The dotted line represents an alternative model ignoring any intervention dates (i.e., days 31, 39, 43 and 46) during the first wave. The dashed line represents another alternative model ignoring intervention dates (days 162 and 165) during the second wave.

**Figure A1:**
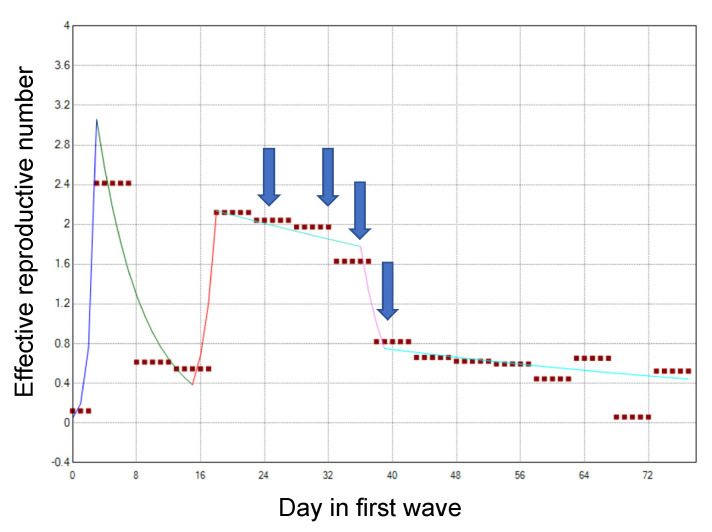
Joinpoint analysis during the first wave of coronavirus disease 2019 in Osaka, Japan. Closed squares indicate the effective reproductive number. The colored line indicates the regression line linking identified joinpoints. Blue arrows represent starting days of the four key interventions during the first wave. Day 0 corresponds to 24 February 2020.

The results of multiple model comparisons are shown in Appendix A Table A1 and Table A2. Fifteen different models for the first wave were compared against the full model. Among them, nine models fitted better than the full model; in these models, two or more event dates were removed, along with either or both of day 43 and day 46. Models that removed only a single event date did not differ significantly from the full model. Using Holm’s method rather than Bonferroni correction did not alter our conclusions (results not shown). Figure 4 compares models using 5-day period estimates of *R*(*t*) against event-based models of *R*(*t*). The full model qualitatively captured estimates of *R*(*t*), except on dates with limited numbers of cases and broad uncertainty bounds of *R*(*t*). When four important events during the first wave were ignored, the model fit was significantly poorer (likelihood ratio test *p*-value = 0.0006). Joinpoint analysis showed that days 43 and 46 were inflection points marking changes in the trend of *R*(*t*) (Appendix A Table A1 and Figure A1). The relative reproduction numbers following days 31, 39, 43 and 46 compared with values prior to day 31 were 1.56 (95% CI: 0.83, 2.81), 0.86 (95% CI: 0.33, 2.01), 0.79 (95% CI: 0.21, 2.63) and 0.32 (95% CI: 0.12, 1.09), respectively. Although the estimates of the single-day effect for days 39, 43 and 46 were all below 1, the upper boundaries of the 95% CIs of all single-day estimates exceeded 1. Combining the effects of all four events, the relative reproduction number was estimated at 0.34 (95% CI: 0.19, 0.59).

**Figure A2:**
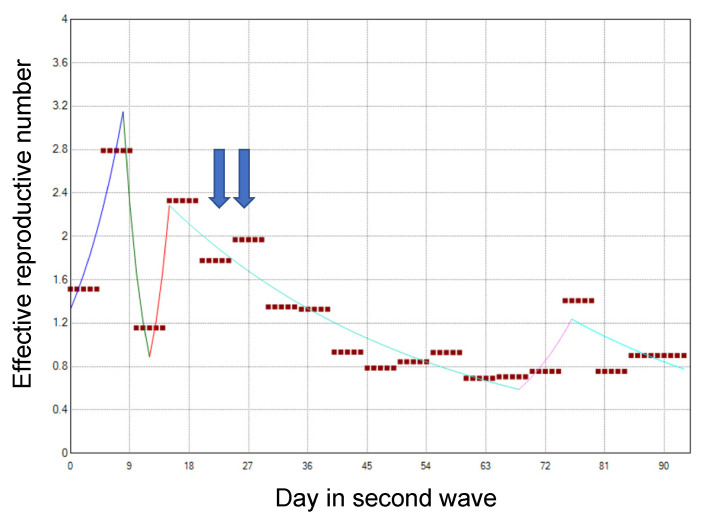
Joinpoint analysis during the second wave of coronavirus disease 2019 in Osaka, Japan. Closed squares indicate the effective reproductive number. The colored line indicates the regression line linking identified joinpoints. Blue arrows represent starting days of two key interventions during the second wave. Day 0 corresponds to 16 June 2020.

The second wave involved two event dates (day 162 and 165). Removing either or both events did not produce significant differences compared with the full model (Appendix A Table A2). Neither of these two event dates was identified as a joinpoint (Appendix A Table A2 and Figure A2). The relative reproduction numbers following days 162 and 165 compared with values prior to day 162 were 0.57 (95% CI: 0.13, 1.54) and 0.87 (95% CI: 0.28, not calculable), respectively. Again, the estimates of the single-day effect were below 1, but the upper boundaries of the 95% CIs exceeded 1 or were not calculable. Combining the effects of these two dates, the relative reproduction number was 0.49 (95% CI: 0.28, 0.79).

When the 5-day model was employed, the *R*(*t*) reached <1 for the first time on 2 April. Alternatively, employing 4-day and 3-day period models, the *R*(*t*) reached <1 for the first time on 1 and 2 April, respectively. Using the 5-day model, the *R*(*t*) declined to <1 from 26 July. Alternatively, using the 4-day and 3-day period models, the *R*(*t*) declined to <1 from 26 and 28 July.

## 4. Discussion

The present study proposed a method for computing the *R*(*t*) of COVID-19. We applied this method to the epidemic in Osaka prefecture from February to September 2020, which comprised two distinct waves. We employed a modified renewal equation based on the date of illness onset, leveraging the frequency of secondary transmission to capture the unique characteristics of COVID-19 (i.e., pre-symptomatic transmission). Using a piecewise constant model with a 5-day time interval to estimate *R*(*t*), we showed that the epidemic came under control around 2 April during the first wave and around 26 July during the second wave. The estimates from our model agreed well with those from an established method using the back-calculated incidence of infection. By allowing *R*(*t*) to be described by the date of infection, we demonstrated that *R*(*t*) did not decline drastically following any single PSHM event. Rather, when multiple interventions were combined, the relative reductions in *R*(*t*) during the first and second waves were 70 and 51%, respectively. While interventions during the second wave were focused on high-risk groups, our model comparison indicated that the combined effect of interventions during the second wave did not have a significant impact on the observed secondary transmission patterns. Because our estimates reflected the impacts of interventions, estimates of *R*(*t*) calculated using the modified renewal equation can be used to assess the effectiveness of PHSMs in a retrospective manner.

The pragmatic value of estimating *R*(*t*) lies in the objective monitoring of epidemiological dynamics in real-time, as well as in assessing the effectiveness of PHSMs by observing their impact on *R*(*t*). The COVID-19 pandemic has catalyzed a flurry of studies that statistically estimate *R*(*t*). A technical debate has emerged with regard to preferred methods for the estimation of *R*(*t*) depending on practical public health needs (e.g., real-time or short-term monitoring of epidemics, forecasting of future trends, and assessing the impacts of interventions) [14,27,28]. We found that the *R*(*t*) did not abruptly decline following single PHSM events. Rather, the combined effects of multiple interventions reduced the *R*(*t*) to <1. During the first wave, various forms of self-restraint in reducing contacts were called for. The combined effect was to reduce the *R*(*t*) by 70%; in fact, the *R*(*t*) declined to <1 when individuals were asked to abstain from night life and reduce contacts in high-risk settings (e.g., eating and drinking behaviors). Joinpoint analysis also suggested that a new trend of reduced *R*(*t*) occurred following the implementation of these two interventions. During the second wave, we found that even without declaring a state of emergency, the *R*(*t*) declined to <1 and the combined effect of interventions was to reduce *R*(*t*) by 50%. However, our model comparison indicated that removing intervention dates during the second wave did not significantly alter the model fit. None of the starting dates of interventions were identified as inflection points of trends in the *R*(*t*) in joinpoint analysis, in contrast with the findings for the first wave. Thus, the impact of interventions, including requests for self-restraint in avoiding eating and drinking with five or more people, on the observed transmission dynamics was limited. Our model also revealed humped patterns of *R*(*t*) with significant variance soon after the ending of major restrictions on 21 May, indicating that *R*(*t*) estimates reflect the cessation of interventions such as requests for self-restraint behaviors. Infections did not continue to increase on and after the hump on 31 August, perhaps because self-restraint in reducing contact behaviors persisted even after the end of restrictions in restaurants.

Our proposed method could accurately estimate *R*(*t*) using illness onset data. *R*(*t*) as a function of time of infection is essential to evaluate intervention programs [2,3]. To date, such evaluations have required non-parametric back-projection of the time of infection using the observed time of illness onset [2]. Unfortunately, non-parametric back-projection is not a simple procedure, sometimes involving expectation-maximization techniques and smoothing [25], and it is undesirable to use two different inferential steps (estimation of the epidemic curve by the time of infection and subsequent estimation of *R*(*t*)). Compared with the procedure involving multiple inferential steps, the advantage of our method is the simplicity of the estimation procedure while accounting for a critical element of the natural history of COVID-19 (i.e., pre-symptomatic transmission). The basis of our strategy was to decompose the data generation process into two components: (i) the relative frequency of secondary transmission with respect to disease-age, and (ii) the probability density function of the incubation period. That is, by decomposing the generation time into two components (i.e., (i) and (ii)) and taking the double integral of the renewal equation, the proposed method enabled us to estimate *R*(*t*) as a function of the time of infection only by using the time of illness onset data. The derivation process is simple (as described in Equations (1) to (4)), and the *R*(*t*) estimates were consistent with those obtained using conventional methods. A small difference was that we employed a step function for *R*(*t*) to avoid complex smoothing procedures; this function can be varied and improved flexibly, and by employing a spline function, far smoother estimates of *R*(*t*) than ours can be obtained. Our method can be applied to other infectious disease datasets if the probability density function of the incubation period and the relative frequency of secondary transmission, with respect to disease-age, are both known and quantified. One such example is smallpox (Nakajo and Nishiura, submitted).

To assess the relationships between intervention timing and *R*(*t*), several different statistical techniques can be used. Potential methods include, but are not limited to, (i) permutation methods (e.g., synthetic control methods that have recently been applied to COVID-19 [29]), (ii) time series methods such as vector autoregression models, and (iii) model comparisons using penalized log likelihood values. We chose option (iii) because the number of important events to be examined was limited, and it was not feasible to select appropriate controls in other prefectures where single PHSMs or combinations of PHSMs were implemented during the period of our analysis. The advantages of model comparison include the ability to account for the degree of freedom (i.e., the number of parameters to be estimated) and the capacity to examine if events are essential to describe epidemic dynamics. However, model comparison cannot be applied when there are too many candidate dates on which trends changed (in these circumstances, permutation would be preferred), and it cannot examine causal links between time events and transmission (in these circumstances, vector autoregression may be preferred). Employing model comparison, we were able to demonstrate that combinations of interventions (but not single interventions) during the first wave were critical in describing transmission dynamics. Intervention dates during the second wave were not significant in describing transmission dynamics (perhaps because of the reduced level of *R*(*t*) during the second wave). Thus, infection control policy should not rely on single interventions that focus on high-risk groups. The combined effect in reducing *R*(*t*) during the second wave was estimated as the product of the effects of each PHSM event, and was as large as a 50% reduction. However, the effect of combined interventions during the second wave was not significant, following Bonferroni correction for multiple testing. Joinpoint analysis also identified significant changes in reduced *R*(*t*) corresponding to the beginnings of interventions during the first wave, but not during the second wave.

Our estimation of the relative reduction in *R*(*t*) indicated that the effect of each intervention event during the first wave was limited, with the upper bounds of relative reductions exceeding the value of *R*(*t*) = 1. The finding is consistent with the results of model comparison, implying that each single intervention was insufficient in reducing *R*(*t*), but that the combined effects of multiple interventions, perhaps coupled with behavioral changes in the population, were required to alter transmission dynamics. The combined effects of multiple interventions during both the first and second waves significantly and substantially reduced the *R*(*t*) to <1.

Our study had several limitations. First, the heterogeneity of transmission was ignored in our model. Specifically, several studies have suggested that age could be a modifying variable in terms of secondary transmission [30,31], so the impact of age structure or occupation, or both, on *R*(*t*) should be explored to characterize the COVID-19 epidemic more accurately. Second, as is the case for other methods, we were unable to estimate *R*(*t*) for recent calendar time-points. This limitation relates to reporting delays as well as the natural history of the disease. We assumed that pre-symptomatic transmission could occur 12 days prior to illness onset at the earliest, as suggested by He et al. [18]. Thus, we could not estimate *R*(*t*) for the most recent 12 days, which may be an issue for assessing the epidemic in real-time. Third, an important technical caveat is that we assumed that transmission potential relative to disease-age was independent of calendar time, which may not be true. A published report has shown that the serial interval of COVID-19 decreased throughout the epidemic [32], suggesting that the assumption of a stable distribution for the serial interval may not be valid. Other key parameters, including frequency of secondary transmission, might also depend on calendar date, and this is an ongoing area of focus for future studies [33]. The proportion of symptomatic transmission could also be modified by interventions targeting manifestations of symptoms (e.g., case isolation and contact tracing) over the course of the epidemic. Fourth, stochasticity during the transmission process was not considered in our model. Fifth, the estimation of *R*_0_ and *R*(*t*) is recognized as subject to high variability using limited empirical data [34].

Despite these technical limitations, the present study was successful in estimating the *R*(*t*) for COVID-19 over the course of the epidemic in Osaka prefecture from February to September 2020. The *R*(*t*) of COVID-19 did not decline following a single intervention event, and our results indicate that concerted efforts would be required to curb the COVID-19 epidemic. The outcome of the COVID-19 pandemic continues to rely on political leadership to swiftly design and implement combined interventions to broadly and appropriately reduce contacts, especially before transmission extends from high-risk settings to the broader community.

## Figures and Tables

**Table 1:** Chronology of the coronavirus disease 2019 (COVID-19) epidemic in Osaka.

Calendar Date	Analysis Date	Description
First wave		
17 February	Day 0	The index case developed illness.
1 March	Day 13	The governor requested the government to dispatch an emergency operations center team (“cluster busters team”).
19 March	Day 31	Voluntary restrictions on crossing the border of Osaka (especially between Osaka and Hyogo) were requested.
27 March	Day 39	Voluntary restrictions on weekend outings were requested.
31 March	Day 43	Voluntary restrictions on eating at restaurants operating at nighttime were requested.
3 April	Day 46	Voluntary restrictions on weekend outings were requested for the second time.
7 April	Day 50	A state of emergency was declared by the Prime Minister.
5 May	Day 78	The original classification of the epidemiological situation (the “Osaka model”) was announced.
21 May	Day 94	Osaka was released from the state of emergency.
Second wave		
12 July	Day 146	A first alert was issued according to the Osaka model.
28 July	Day 162	Voluntary restrictions on social events involving the consumption of alcohol by more than four persons were requested.
31 July	Day 165	Restaurants and bars in specific districts of Osaka city were requested to voluntarily close.
21 August	Day 186	Restaurants and bars were allowed to open.
31 August	Day 196	Restrictions on social events involving the consumption of alcohol by more than four persons were lifted.

## Data Availability

The epidemiological data analyzed in this study are publicly available (https://covid19-osaka.info/; Accessed on 17 March 2021) and downloadable as the online supplementary data of this study.

## Supplementary Materials

The following are available online at https://www.mdpi.com/2077-0383/10/6/1256/s1.

## Appendix A

**Table A1:** Multiple comparisons of the effective reproduction number accounting for the absence of dates of specific event of interventions and the identification of joinpoints during the first wave of coronavirus disease 2019 in Osaka, Japan.

Model	Day 31	Day 39	Day 43	Day 46	*p*-Value	
1	0	1	1	1	0.24059	
2	1	0	1	1	0.76617	
3	1	1	0	1	0.67850	
4	1	1	1	0	0.15528	
5	0	0	1	1	0.36975	
6	0	1	0	1	0.40291	
7	0	1	1	0	0.00000	*
8	1	0	0	1	0.00000	*
9	1	0	1	0	0.00000	*
10	1	1	0	0	0.00000	*
11	0	0	0	1	0.00000	*
12	0	0	1	0	0.00000	*
13	0	1	0	0	0.00000	*
14	1	0	0	0	0.00000	*
15	0	0	0	0	0.00059	*
joinpoint	N	N	Y	Y		

Model column gives the ID of each single combination of days on which interventions were initiated. * Statistically significant following Bonferroni correction (*p* < 0.00333). A value of 0 indicates that the corresponding model ignored the corresponding date, whereas 1 indicates that the date was taken into account. *p*-values show the results from likelihood ratio tests compared against the full model. Y/N indicates whether a joinpoint was identified or not within the 5-day period including the corresponding day.

**Table A2:** Multiple comparisons of the effective reproduction number accounting for the absence of dates of specific events of interventions and the identification of joinpoints during the second wave of coronavirus disease 2019 in Osaka, Japan.

Model	Day 162	Day 165	*p*-Value
1	0	1	0.45741
2	1	0	0.66792
3	0	0	0.86868
joinpoint	N	N	

Model column gives the ID of each single combination of days on which interventions were initiated. A value of 0 indicates that the corresponding model ignored the corresponding date, whereas 1 indicates that the date was taken into account. *p*-values show the results from likelihood ratio tests compared against the full model. Y/N indicates whether a joinpoint was identified or not within the 5-day period, including the corresponding day.
